# Supraorbital transcutaneous neurostimulation has sedative effects in healthy subjects

**DOI:** 10.1186/1471-2377-11-135

**Published:** 2011-10-28

**Authors:** Maxime Piquet, Costantino Balestra, Simona L Sava, Jean E Schoenen

**Affiliations:** 1 Environmental, Occupational and Ageing Physiology Laboratory, DAN Europe Research, Haute Ecole Paul Henri Spaak, I.S.E.K., Brussels, Belgium; 2 Headache Research Unit. Department of Neurology & GIGA - Neurosciences. Liège University, CHU-Sart Tilman. T4 (+1). B36., B-4000 LIEGE. Belgium

## Abstract

**Background:**

Transcutaneous neurostimulation (TNS) at extracephalic sites is a well known treatment of pain. Thanks to recent technical progress, the Cefaly^® ^device now also allows supraorbital TNS. During observational clinical studies, several patients reported decreased vigilance or even sleepiness during a session of supraorbital TNS. We decided therefore to explore in more detail the potential sedative effect of supraorbital TNS, using standardized psychophysical tests in healthy volunteers.

**Methods:**

We performed a double-blind cross-over sham-controlled study on 30 healthy subjects. They underwent a series of 4 vigilance tests (Psychomotor Vigilance Task, Critical Flicker Fusion Frequency, Fatigue Visual Numeric Scale, d2 test). Each subject was tested under 4 different experimental conditions: without the neurostimulation device, with sham supraorbital TNS, with low frequency supraorbital TNS and with high frequency supraorbital TNS.

**Results:**

As judged by the results of three tests (Psychomotor Vigilance Task, Critical Flicker Fusion Frequency, Fatigue Visual Numeric Scale) there was a statistically significant (p < 0.001) decrease in vigilance and attention during high frequency TNS, while there were no changes during the other experimental conditions. Similarly, performance on the d2 test was impaired during high frequency TNS, but this change was not statistically significant.

**Conclusion:**

Supraorbital high frequency TNS applied with the Cefaly^® ^device decreases vigilance in healthy volunteers. Additional studies are needed to determine the duration of this effect, the underlying mechanisms and the possible relation with the stimulation parameters. Meanwhile, this effect opens interesting perspectives for the treatment of hyperarousal states and, possibly, insomnia.

## Background

Neurostimulation is a therapeutic method where action potentials are elicited by depolarizing nerve fibres with electrical impulses produced by a current generator device generally called neurostimulator. This method is used percutaneously with implantable neurostimulators and electrodes positioned over the spinal cord or peripheral nerves, or transcutaneously via superficial skin electrodes and external neurostimulators.

Percutaneous neurostimulation (PNS) of the spinal cord has been developed in the last decade for the management of intractable pain [1,2], but also for the treatment of several neurological disorders such as spasticity [3], parkinsonian tremor [4] or epilepsy [5], More recently, PNS has been explored for the treatment of intractable headaches [6-11].

Transcutaneous neurostimulation (TNS) is a classical technique which has demonstrated its efficacy in the treatment of pain [12,13] and is nowadays largely in use in pain clinics and physical therapy centres. It has the advantage of being non-invasive, safe and almost devoid of adverse effects contrary to PNS which needs a surgical intervention to implant the electrodes and the neurostimulator.

TNS at cephalic sites has been technically difficult and usually rather painful. STX-Med company has recently developed a headset for TNS of supratrochlear and supraorbital nerves, both branches of the ophthalmic division of the trigeminal nerve (V1), making the technique comfortable and easy to use [14]. Consequently, the utility of TNS in the treatment and prevention of headaches and migraine has been investigated [15] and several clinical trials are underway. Subjects enrolled in those trials have repeatedly reported that supraorbital TNS tended to affect vigilance and decrease attention with a tendency to fall asleep during the stimulation.

Cephalic electrical stimulation has been used many years ago to induce sleep or decrease anxiety. The method known as "Cranial Electrotherapy Stimulation (CES)", also called transcranial or transcerebral electrostimulation differs from TNS in that its objective is to generate different types of electrical currents through the head and not to specifically stimulate cranial nerves like TNS. For this purpose, CES uses generally an anterior frontal or a jaw electrode and a posterior electrode placed over the mastoid process [16,17]. CES was reported to have some effects on anxiety, depression and insomnia [18-20].

Given the anecdotal reports by patients of TNS-induced sedative effects, not hitherto reported in the literature, and the reported mental effects of CES, we decided to explore the effect on vigilance of supraorbital TNS with the headset developed by STX-Med in a double blind cross-over study.

## Methods

We performed a double-blind crossover sham-controlled study of 30 subjects to assess the effect on vigilance of different protocols of supra-orbital TNS. Each subject was tested in 4 different experimental conditions: without neurostimulation device (blank control: BC), with a sham neurostimulation (Sham control: SC), with a low frequency neurostimulation (LFN) and with a high frequency neurostimulation (HFN). The study protocol was approved by the local ethics committee (CE B200-2010-074-2010-05-03).

### Subjects

We included 30 healthy subjects: 15 men and 15 women ranging in age from 19 to 29 years (mean age = 23,9 +/- 2.4).

To be eligible, subjects had to be right-handed, drink no more than 1 cup of tea or coffee per day and no more than 2 glasses of alcohol per week. Exclusion criteria were a history of serious surgical, medical or psychiatric disease, smoking, and drug intake. Informed consent was obtained for all subjects prior to the study.

### Neurostimulation

**Figure 1:**
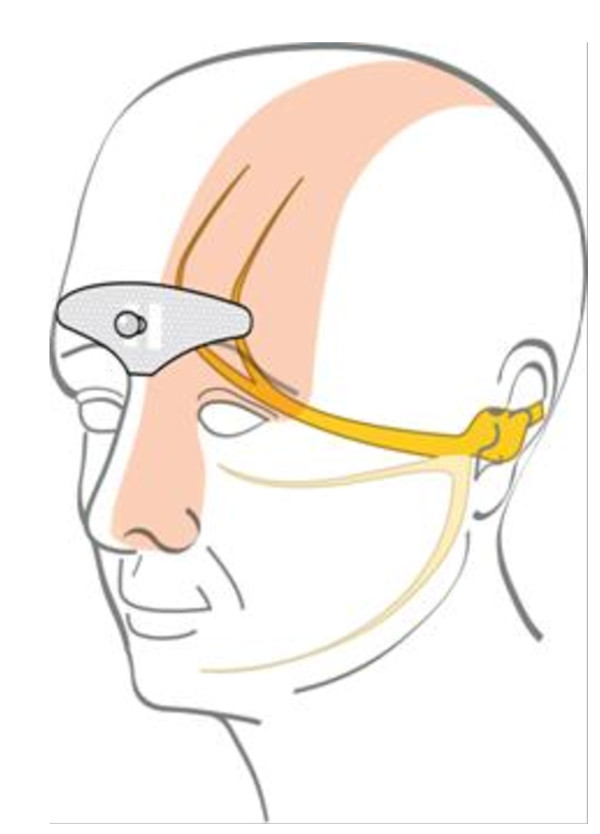
**The stimulation electrode placed on the forehead covers the supratrochlear and supraorbital nerves**.

Supra-orbital neurostimulation was delivered with an external self adhesive electrode placed on the forehead (see Figure 1). The bipolar electrode is designed in order to cover the supratrochlear and supraorbital nerves bilaterally. Its dimensions are 30 mm × 94 mm.

The neurostimulator was a Cefaly^® ^device (STX-Med, Liège, Belgium). It is a constant current generator for a maximum skin impedance of 2.2 KΩ. It generates biphasic rectangular impulses with an electrical mean equal to zero. The impulses have the following parameters: impulse width 250 μS, maximum intensity 14 mA. Low frequency neurostimulation (LFN) was delivered at a frequency of 2.5 Hz, high frequency neurostimulation (HFN) at 120 Hz. The neurostimulation lasted 20 minutes. For both LFN and HFN, the intensity reached was above perception threshold, so that all subjects experienced paresthesias and tingling under the stimulation electrodes. For sham neurostimulation (SC) we used a Cefaly^® ^device with a low current intensity of 1 mA that was below the perception threshold and produced no sensation detectable by the subjects.

### Psychophysical measures

Four psychophysical tests were selected to detect sedative effects.

1) The *Psychomotor Vigilance Task *(PVT) was developed [21] to measure performance during mental fatigue. It is regarded as the gold standard for sleepiness.

We used the PEBL [22] implementation of the PVT (PPVT). Briefly, the subject sits in front of a black computer screen. As soon as a red dot appears, the subject is supposed to hit the space bar of the computer keyboard. The reaction time is recorded in milliseconds. In total 12 reaction times measures are measured for each PVT test, separated randomly by intervals of 2 to 12 seconds. The results are expressed as the mean value of the 12 measures.

2) The *Critical Flicker Fusion Frequency *(CFFF) test is defined as "the highest or lowest temporal frequency, at a given percentage modulation, that can be resolved" [23], i.e. the frequency at which the subject is able to distinguish a flashing from a steady light. The CFFF decreases with fatigue. A portable device powered with a 9 V battery and equipped with a blue LED was used to vary flicker frequency by 0.5 Hz steps. The device starts with a steady light and the flicker frequency is decreased until the subject reports that the light is flashing. This frequency is recorded as the CFFF for that experiment.

3) The *d2 test *for attention and concentration [24] allows to assess visual attention and the ability to concentrate on a task. It consists of 14 lines of a combination of the letters "d" and "p" with one to four dashes placed above and/or below the letter. The objective is to mark all "d" with two dashes within 20 seconds for each line. Three scores are evaluated: GZ ("Gesamtzahl der bearbeiteten Zeichen") is the total number of letters marked; KL ("Konzentrationsleistungswert") is the number of correct letters marked minus the number of non correct letters; and F% ("Fehlerprozentwert") representing the percentage of errors compared to the number of characters marked (GZ). As this test can be biased by a learning effect, it is only presented once during the session without recording of a baseline.

4) For the subjective evaluation of fatigue we used the *Fatigue Visual Numeric Scale *(FVNS - Stanford Patient Education Research Centre [25]. This is a visual analogue scale where the subject scores fatigue from 0 (not tired at all) to 10 (very tired).

### Procedures

Two groups of 8 subjects and two groups of 6 groups performed the experiments as depicted in Table 1. The sessions were separated by at least 6 hours as to ensure there was no remaining effect of the stimulation.

**Table 1:** Schedule of the experiments for each group

	First Experiment	Second Experiment	Third Experiment	Fourth Experiment
**Group I**	Tuesday 8 AM	Tuesday 2 PM	Thursday 8 AM	Thursday 2 PM

**Group II**	Tuesday 9 AM	Tuesday 3 PM	Thursday 9 AM	Thursday 3 PM

**Group III**	Tuesday 10 AM	Tuesday 4 PM	Thursday 10 AM	Thursday 4 PM

**Group IV**	Tuesday 11 AM	Tuesday 5 PM	Thursday 11 AM	Thursday 5 PM


At the first session, each subject of the group is randomly assigned to one of the 4 experimental conditions:

• LFN, where the subjects get a Low Frequency Neurostimulation

• HFN, where the subjects get a High Frequency Neurostimulation

• SC, where the subjects get a sham neurostimulation (Sham Control)

• BC, where the subjects do not have a device (Blank Control)

Two subjects are assigned to each condition. In the subsequent sessions, the same subjects are re-assigned to another condition in order for each of them to have been through each condition after the 4 sessions.

The subjects are sitting comfortably in a chair in front of a wall to avoid any distraction. Once the session has started, each subject fills in the FVNS and performs the PPVT test where after the CFFF is determined. After these baseline tests, the neurostimulation is started for all subjects assigned to conditions LFN, HFN and SC while no neurostimulation is applied for the subjects assigned to condition BC. After 10 minutes of stimulation for LFN, HFN and SC or a 10-minute waiting time for BC, the subjects perform the d2 test that lasts 280 s. Thereafter they score FVNS once more, redo the PPVT test and finally have the CFFF measured again. The psychophysical tests are thus studied in the same sequence under every experimental condition.

This means in practice that we have a set of results for FVNS, PPVT and CFFF as measured before the application of the neurostimulator. A second set of results is obtained while the neurostimulator is applied since ± 15 minutes. The results can therefore also be expressed as a percentage of the measurement during the neurostimulation compared to the baseline value recorded before the neurostimulation.

### Statistical Analysis

We compared the results of the psychophysical tests for each of the 4 experimental conditions: LFN, HFN, SC and BC. For FVNS, PPVT and CFFF we used the variation in percentage between pre- and perstimulation values to verify the effects of the 4 conditions. Since the results did not have a Gaussian distribution, we used the Wilcoxon test to measure the significance of the variation observed.

For the d2 test, we compared GZ, KL and the F% between the 4 conditions (as there was no control values to compare with). We have used the Mann-Whitney test to verify the significance of the differences observed.

## Results

### PPVT Test

**Figure 2:**
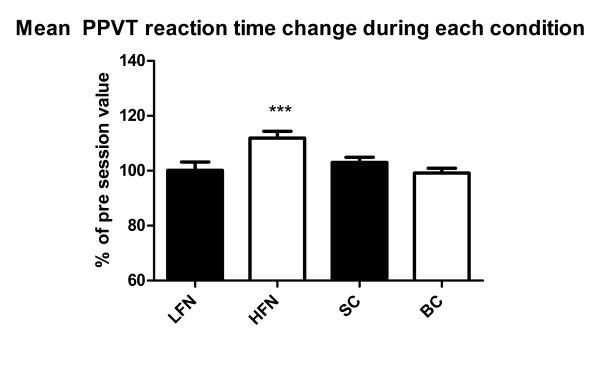
Mean PPVT reaction time change during the experimental conditions expressed as a percentage of the baseline value (*** = p < 0.001) (mean+/-SEM).

The mean reaction times (RT) for the PPVT (N = 30) before the session was 339 ms+176 for LFN, 304 ms + 37 for HFN, 294 ms + 44 for SC and 306 ms + 46 for BC. Reaction time increased during HFN, while it was stable for the LFN, SC and BC conditions (Figure 2).

As explained in the methods section, for FVNS, PPVT and CFFF the statistical analysis was performed on the ratio (in percentage) between the mean value during and before the experimental condition for each subject. The mean percentage increase in RT is significant only during the HFN condition (p = 0.0002).

### CFFF Test

**Figure 3:**
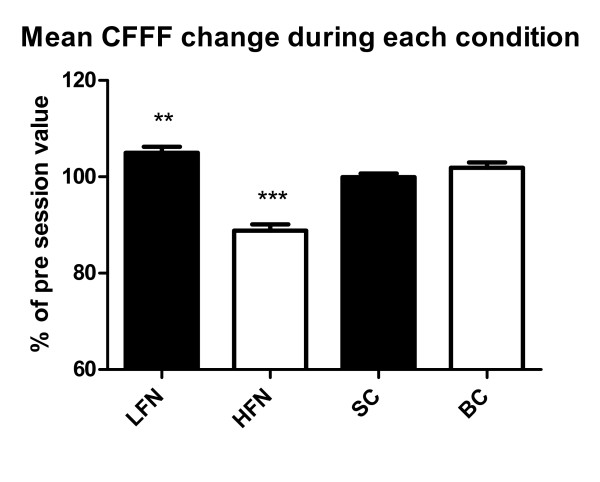
Mean CFFF change during the experimental conditions expressed as a percentage of the baseline value (** = p < 0.01; *** = p < 0.001) (mean+/-SEM).

The mean values for CFFF (N = 30) before the session was 38.2 Hz + 2.5 for LFN, 39.7 Hz + 2.7 for HFN, 39.9 Hz + 3.3 for SC and 38.2 Hz + 2.2 for BC. During HFN there was a significant decrease of CFFF (p < 0.0001) while CFFF was significantly increased during LFN (Figure 3).

### d2 Test

Table 2 shows the results for the d2 test. Mean values of GZ, KL and F% are given during each experimental condition the. Numerically the total number of letters marked (GZ) and the number of correct letters marked (KL) were the lowest in the HFN condition, while the percentage of errors was the highest, but this difference was not statistically significant.

**Table 2:** d2 results.

N = 30	LFN	HFN	SC	BC
Mean value of GZ	560 ± 77	544 ± 80	587 ± 57	562 ± 70
Mean value of KL	215 ± 40	214 ± 50	229 ± 42	217 ± 43
Mean value of F%	6.95% ± 6.81	8.37% ± 8.38	6.02% ± 5.98	6.72% ± 6.16

### Fatigue Visual Numeric Scale

**Figure 4:**
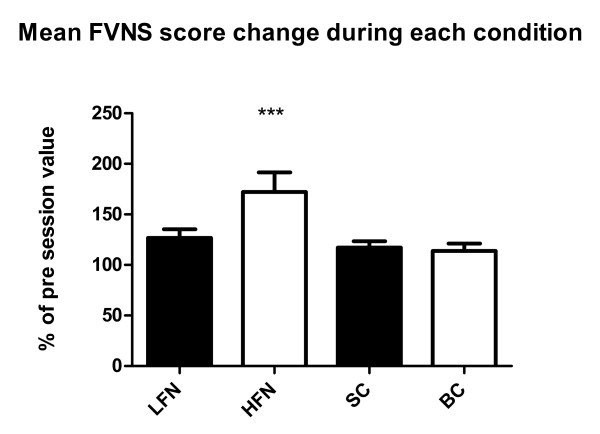
Mean change in FNVS score during the experimental conditions expressed as a percentage of the baseline value (*** = p < 0.001) (mean+/-SEM).

The FVNS fatigue score tended to increase during all four conditions. However, the statistical analysis for the averaged individual changes showed that the increase was significant only during HFN (Figure 4).

## Discussion

Taken together our results suggest that supraorbital neurostimulation using the Cefaly^® ^device decreases arousal and induces fatigue. This cannot be considered at this stage as a hypnotic effect in the sense of inducing sleep and decreasing sleep latency but rather as a sedative effect in terms of a reduction of alertness and vigilance. Interestingly, this is only the case with high (120 Hz-HFN) and not with low frequency (2.5 Hz-LFN) stimulation. LFN even has an opposite effect in one psychophysical test, the critical flicker fusion frequency. Below we will examine these results in more detail and speculate on possible mechanisms.

The Psychomotor Vigilance Task measures the reaction time (RT) and is considered as the gold standard for measuring sleepiness [21]. That it is readily reproducible is demonstrated by the fact that during the blank condition (BC) the change compared to baseline was less than 1.5%. Sham (SC) and LFN induced non significant increases in RT of respectively 8.9 ms and 8.6 ms. By contrast, HFN increased RT by an average of 36.7 ms, i.e. by more than 10%. Critical flicker fusion frequency is known to decrease with fatigue. While unchanged during SC and minimally increased during BC (+ 0.9 Hz), it increased during LFN (+ 1.9 Hz) possibly suggesting a mild increase in vigilance. Again HFN contrasted with all other conditions by a marked decrease (-4.6 Hz) in CFFF, indicating a decrease in arousal. This result is concordant with that of the subjective fatigue rating on the Fatigue Visual Numerical Scale (FVNS). The subjects rated their fatigue higher during all experimental conditions than at baseline, which was not significant and might be related to the mental strain due to the recordings or to a learning effect in using the numerical scale. However, the increase of the FVNS score during HFN was three times greater (+ 72.1%). The d2 test for attention and concentration was in our study the only one for which the HFN condition induced no significant effect. Nevertheless the numerical changes during HFN are in line with the other results as they show a lower number of total letters marked and of correct letters marked as well as a higher number of errors. The lack of significance could have at least two explanations. First, the d2 test was administered at an earlier time point (between 10 and 15 minutes) during the experimental condition compared to the other tests (from 15 minutes onwards). The duration of HFN might thus not have been long enough to produce significant d2 test changes. Second, this test was performed only once to avoid a learning effect and the pre- and per-condition comparison had therefore to be replaced by a comparison between conditions, hence weakening the sensitivity of the test to detect a change.

To the best of our knowledge, this is the first time that the effect of transcutaneous neurostimulation on arousal and fatigue was studied in humans and there are no similar studies available in animals. The neurobiological mechanisms through which HFN induces sedation remain therefore speculative. Some insight can nonetheless be gained from the studies of transcutaneous neurostimulation in Alzheimer's patients and from those in experimental animals of the central nervous system consequences of electroacupuncture. A Dutch group reported in a series of publications that transcutaneous electrostimulation was able to improve memory, alertness [26,27] and rest-activity rhythm [28] in Alzheimer's disease. This effect was attributed to activation of the hippocampus and the suprachiasmatic nucleus both by direct spinal cord afferents [29] and via the dorsal raphe nucleus and locus coeruleus [30,31]. Although vigilance was not specifically measured in these studies, the observed cognitive and behavioural effects would suggest increased arousal and vigilance rather than sedation like in our study. This opposite effects can probably be explained by the different stimulation protocols. First, Alzheimer patients received transcutaneous neurostimulation over paravertebral back muscles daily during 6 [26] or 3 hours [27,28] for 6 weeks while we used a single 20-minute session of supraorbital neurostimulation. In a more recent randomized sham-controlled pilot trial of right median nerve stimulation, Scherder et al [32] found no significant effect on memory in Alzheimer's disease and the same group reported that cranial electrostimulation had no effect on rest-activity rhythm neither at low frequency [33] nor at high frequency [34]. More interestingly, we found a hypnotic effect with high frequency (120 Hz) stimulation, whereas the beneficial effects in Alzheimer's disease were obtained with burst of stimuli (9 pulses at 160 Hz) delivered at a low frequency of 2 Hz, a frequency that in our study concordantly increased critical flicker fusion frequency. One may assume that high and low frequency stimulations can have different effects on central nervous system structures and thus on arousal, but this remains to be proven in an adequate study.

Transcranial direct current stimulation (tDCS) is able to modulate cortical activity under certain conditions and in certain brain areas. It is extremely unlikely, however, that the supraorbital TNS used in this study influences directly the underlying brain structures, i.e. the frontal lobes, for at least two reasons. First, The small electrode surface (7 cm²) and distance between the two electrodes (5 mm) restrict the skin surface affected by the current as well as current penetration into deeper structures. Second, the TNS applied current is composed of biphasic rectangular impulses with an electrical mean equal to zero, while tDCS uses a direct current. The current characteristics and the mechanisms of action are thus different between trigeminal TNS and tDCS. Moreover, in a recent study [35], weak transcranial electrical DC or AC currents over the prefrontal cortex had no effect on mood or EEG in healthy subjects. Interestingly, sleepiness was reported rarely both in the active (0.11%) and sham stimulation groups (0.08%).

Experimental studies on the mode of action of electroacupuncture in pain are relevant to this discussion because many of the central nervous system structures activated by electroacupuncture like the monoaminergic brain stem nuclei, the hypothalamic arcuate nucleus or the periaqueductal gray matter also play a role in vigilance states (36,37,38,39). A simple straightforward explanation for the sedative effects found in our study would be an effect of the transcutaneous stimulation on monoaminergic brain stem nuclei such as locus coeruleus that receives direct spinal input [40]. The locus coeruleus is also thought to mediate the anti-epileptic effect of high frequency transcutaneous stimulation of the ophthalmic nerve [41]. However, in animals high frequency electroacupuncture was found to increase neuronal activity in brain stem nuclei [36], in particular in dorsal raphe nuclei [37]. Increased activity of these nuclei that belong to the ascending activating reticular system would be associated with increased rather than decreased arousal and vigilance. Electroacupuncture over peripheral nerves also activates the hypothalamic arcuate nucleus in animals [39]. The arcuate nucleus plays a pivotal role in electroacupuncture-induced cardiovascular inhibition [39], but also in vigilance states via its reciprocal connections with orexin-containing lateral hypothalamic neurons and the ventrolateral periaqueductal gray matter (38,42). A change in activity levels of the orexin-arcuate-periaqueductal gray matter circuit could occur during supraorbital neurostimulation and might explain the decrease in vigilance. Future studies of supraorbital neurostimulation coupled to functional cerebral imaging studies could verify this hypothesis. Further studies are also needed to verify whether the sedative effects of HFN as evidenced here by psychophysical tests have electroencephalographic correlates and if they are associated with hypnotic effects such as sleep latency reduction.

## Conclusion

To sum up, we have shown in healthy volunteers that supraorbital high frequency neurostimulation applied with the Cefaly^® ^device modifies concordantly several psychophysical tests in a way that is compatible with decreased vigilance and arousal, while sham stimulation has no effect and low frequency neurostimulation, if anything, tends to increase arousal. The precise mechanisms of action of HFN on the CNS arousal systems are not known and warrant further studies. Meanwhile supraorbital HFN with the Cefaly^® ^device opens interesting perspectives for an adverse effect-free treatment of hyperarousal states, and possibly sleep disorders.

## Competing interests

This study was sponsored by STX-Med, Liège, Belgium.

## Authors' contributions

MP has participated in the design of the study, performed the experiments and provided a draft of the results. CB has participated in the design of the study and in the statistical analysis of the results. SLS has made the literature search and JS has interpreted the results in the light of the available literature data and drafted the final manuscript. All authors read and approved the final manuscript.

## Pre-publication history

The pre-publication history for this paper can be accessed here:

http://www.biomedcentral.com/1471-2377/11/135/prepub

## References

[B1] Richardson RR Siqueira EB Cerullo LJ Spinal epidural neurostimulation for treatment of acute and chronic intractable pain: initial and long term results Neurosurgery 1979 5 3 344 8 10.1227/00006123-197909000-00007 315523

[B2] Kapural L Narouze S Janicki T Mekhail N Spinal cord stimulation is an effective treatment for the chronic intractable visceral pelvic pain Pain Med 2006 7 5 440 3 10.1111/j.1526-4637.2006.00165.x 17014604

[B3] Richardson RR McLone DG Percutaneous epidural neurostimulation for paraplegic spasticity Surg Neurol 1978 9 3 153 5 305668

[B4] Alesch F Pinter MM Helscher RJ Fertl L Benabid AL Koos WT Stimulation of the ventral intermediate thalamic nucleus in tremor dominated Parkinson's disease and essential tremor Acta Neurochir (Wien) 1995 136 1-2 75 81 10.1007/BF01411439 8748831

[B5] Handforth A DeGiorgio CM Schachter SC Uthman BM Naritoku DK Tecoma ES Vagus nerve stimulation therapy for partial-onset seizures: a randomized active-control trial Neurology 1998 51 1 48 55 9674777 10.1212/wnl.51.1.48

[B6] Ahmed HE White PF Craig WF Hamza MA Ghoname ES Gajraj NM Use of percutaneous electrical nerve stimulation (PENS) in the short-term management of headache Headache 2000 40 4 311 5 10.1046/j.1526-4610.2000.00046.x 10759936

[B7] Magis D Allena M Bolla M De Pasqua V Remacle JM Schoenen J Occipital nerve stimulation for drug-resistant chronic cluster headache: a prospective pilot study Lancet Neurol 2007 6 4 314 21 10.1016/S1474-4422(07)70058-3 17362835

[B8] Magis D Schoenen J Neurostimulation in chronic cluster headache Curr Pain Headache Rep 2008 12 2 145 53 10.1007/s11916-008-0027-0 18474196

[B9] Schwedt TJ Occipital nerve stimulation for medically intractable headache Curr Pain Headache Rep 2008 12 1 62 6 10.1007/s11916-008-0012-7 18417026

[B10] Bartsch T Paemeleire K Goadsby PJ Neurostimulation approaches to primary headache disorders Curr Opin Neurol 2009 22 3 262 8 10.1097/WCO.0b013e32832ae61e 19434793

[B11] Reed KL Black SB Banta CJ Will KR Combined occipital and supraorbital neurostimulation for the treatment of chronic migraine headaches: initial experience Cephalalgia 2010 30 3 260 71 19732075 10.1111/j.1468-2982.2009.01996.x

[B12] Wall PD Sweet WH Temporary abolition of pain in man Science 1967 155 758 108 9 10.1126/science.155.3758.108 6015561

[B13] Cruccu G Aziz TZ Garcia-Larrea L Hansson P Jensen TS Lefaucheur JP EFNS guidelines on neurostimulation therapy for neuropathic pain Eur J Neurol 2007 14 9 952 70 10.1111/j.1468-1331.2007.01916.x 17718686

[B14] Muller P Rigaux P Medical and technical Cefaly dossier (Annexe × of 93/42 CEE directive). STX-Med scientific file 2007

[B15] Gérardy PY Fabry D Fumal A Schoenen J A pilot study on supra-orbital surface electrotherapy in migraine Cephalalgia 2009 29

[B16] Schmitt R Capo T Frazier H Boren D Cranial electrotherapy stimulation treatment of cognitive brain dysfunction in chemical dependence J Clin Psychiatry 1984 45 2 60 1 2-3 6363398

[B17] Mignon A Laudenbach V Guischard F Limoge A Desmonts JM Mantz J Transcutaneous cranial electrical stimulation (Limoge's currents) decreases early buprenorphine analgesic requirements after abdominal surgery Anesth Analg 1996 83 4 771 5 8831319 10.1097/00000539-199610000-00020

[B18] Ryan JJ Souheaver GT Effects of transcrerebral electrotherapy (electrosleep) on state anxiety according to suggestibility levels Biol Psychiatry 1976 11 2 233 7 786380

[B19] McKenzie RE Costello RM Electrosleep (Electrical Transcranial Stimulation) in the Treatment of Anxiety, Depression and Sleep Disturbance in Chronic Alcoholics J Altered States of Consciousness 1975 2 2 11

[B20] Bystritsky A Kerwin L Feusner J A pilot study of cranial electrotherapy stimulation for generalized anxiety disorder J Clin Psychiatry 2008 69 3 412 7 10.4088/JCP.v69n0311 18348596

[B21] Wilkinson RT Houghton D Field test of arousal: a portable reaction timer with data storage Hum Factors 1982 24 4 487 93 7129455 10.1177/001872088202400409

[B22] PEBL PEBL: The Psychology Experiment Building Language PEBL 2010

[B23] Schneider C Fulda S Schulz H Daytime variation in performance and tiredness/sleepiness ratings in patients with insomnia, narcolepsy, sleep apnea and normal controls J Sleep Res 2004 13 4 373 83 10.1111/j.1365-2869.2004.00427.x 15560772

[B24] Brickenkamp R D2 test d'Attention Concentrée 2007

[B25] Fatigue Visual Numeric Scale http://patienteducation.stanford.edu/research/vnsfatigue.html

[B26] Scherder EJA Bouma A Steen AM Influence of transcutaneous electrical nerve stimulation on memory in patients with dementia of the Alzheimer type J. Clin. Exp. Neuropsychol 1992 14 6 951 960 10.1080/01688639208402546 1360474

[B27] Scherder EJ Bouma A Steen AM Effects of short-term transcutaneous electrical nerve stimulation on memory and affective behaviour in patients with probable Alzheimer's disease Behav Brain Res 1995 67 2 211 9 10.1016/0166-4328(94)00115-V 7779292

[B28] Van Dijk KR Luijpen MW Van Someren EJ Sergeant JA Scheltens P Scherder EJ Peripheral electrical nerve stimulation and rest-activity rhythm in Alzheimer's disease J Sleep Res 2006 15 4 415 23 10.1111/j.1365-2869.2006.00548.x 17118098

[B29] Cliffer KD Burstein R Giesler GJ Distributions of spinothalamic, spinohypothalamic, and spinotelencephalic fibers revealed by anterograde transport of Pha-l in rats J. Neurosci 1991 11 852 868 1705972 10.1523/JNEUROSCI.11-03-00852.1991 PMC6575342

[B30] Hay-Schmidt A Vrang N Larsen PJ Mikkelsen JD Projections from the raphe nuclei to the suprachiasmatic nucleus of the rat J. Chem. Neuroanat 2003 25 293 310 10.1016/S0891-0618(03)00042-5 12842274

[B31] Scherder EJ Luijpen MW van Dijk KR Activation of the dorsal raphe nucleus and locus coeruleus by transcutaneous electrical nerve stimulation in Alzheimer's disease: a reconsideration of stimulation-parameters derived from animal studies Chin J Physiol 2003 46 4 143 50 15074834

[B32] Scherder EJ Vuijk PJ Swaab DF van Someren EJ Estimating the effects of right median nerve stimulation on memory in Alzheimer's disease: a randomized controlled pilot study Exp Aging Res 2007 33 2 177 86 10.1080/03610730701238915 17364906

[B33] Scherder E Knol D van Someren E Deijen JB Binnekade R Tilders F Sergeant J Effects of low-frequency cranial electrostimulation on the rest-activity rhythm and salivary cortisol in Alzheimer's disease Neurorehabil Neural Repair 2003 17 2 101 8 10.1177/0888439003017002004 12814055

[B34] Scherder E Knol D van Tol MJ van Someren E Deijen JB Swaab D Scheltens P Effects of high-frequency cranial electrostimulation on the rest-activity rhythm and salivary cortisol n Alzheimer's disease: a pilot study Dement Geriatr Cogn Disord 2006 22 4 267 72 10.1159/000095108 16912480

[B35] Tadini L El-Nazer R Brunoni AR Williams J Carvas M Boggio P Priori A Pascual-Leone A Fregni F Cognitive, mood, and electroencephalographic effects of noninvasive cortical stimulation with weak electrical currents J ECT 2011 27 2 134 40 10.1097/YCT.0b013e3181e631a8 20938352

[B36] Lee JH Beitz AJ The distribution of brain-stem and spinal cord nuclei associated with different frequencies of electroacupuncture analgesia Pain 1993 52 1 11 28 10.1016/0304-3959(93)90109-3 8446432

[B37] Kwon Y Kang M Ahn C Han H Ahn B Lee J Effect of high or low frequency electroacupuncture on the cellular activity of catecholaminergic neurons in the brain stem Acupunct Electrother Res 2000 25 1 27 36 10830973 10.3727/036012900816356235

[B38] Fort P Bassetti CL Luppi PH Alternating vigilance states: new insights regarding neuronal networks and mechanisms Eur J Neurosci 2009 29 9 1741 53 10.1111/j.1460-9568.2009.06722.x 19473229

[B39] Li P Tjen-A-Looi SC Guo ZL Longhurst JC An arcuate-ventrolateral periaqueductal gray reciprocal circuit participates in electroacupuncture cardiovascular inhibition Auton Neurosci 2010 158 1-2 13 23 10.1016/j.autneu.2010.05.006 20580325 PMC2976778

[B40] Craig AD Spinal and trigeminal lamina I input to the locus coeruleus anterogradely labeled with Phaseolus vulgaris leucoagglutinin (PHA-L) in the cat and the monkey Brain Res 1992 584 1-2 325 8 10.1016/0006-8993(92)90915-V 1515950

[B41] DeGiorgio CM Murray D Markovic D Whitehurst T Trigeminal nerve stimulation for epilepsy: long-term feasibility and efficacy Neurology 2009 72 936 10.1212/01.wnl.0000344181.97126.b4 19273830

[B42] Tsujino N Sakurai T Orexin/hypocretin: a neuropeptide at the interface of sleep, energy homeostasis, and reward system Pharmacol Rev 2009 61 2 162 76 10.1124/pr.109.001321 19549926

